# Supplementary figures and images for: Structure and flexibility of the yeast NuA4 histone acetyltransferase complex
Source: eLife. 2022 Oct 20;11:e81400. doi: 10.7554/eLife.81400 (PMC9643008; doi:10.7554/eLife.81400)

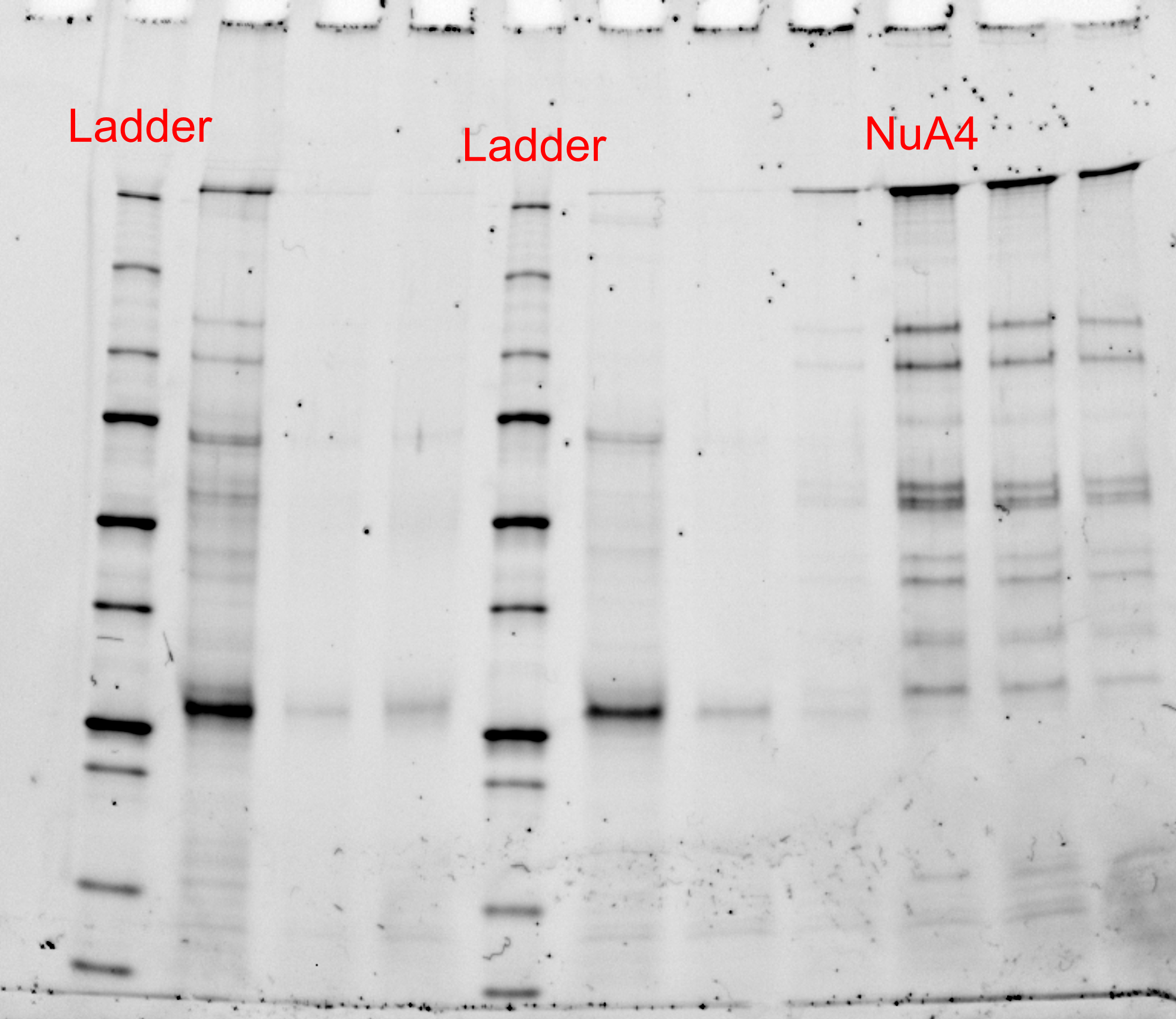

Supplement: Figure 1—figure supplement 1—source data 1. — For panel A. [file elife-81400-fig1-figsupp1-data1.zip › Figure 1-figure supplement 1-source data 1/2017_11_03 NuA4 post_IgG_TEV_CBP [Flamingo].tif]

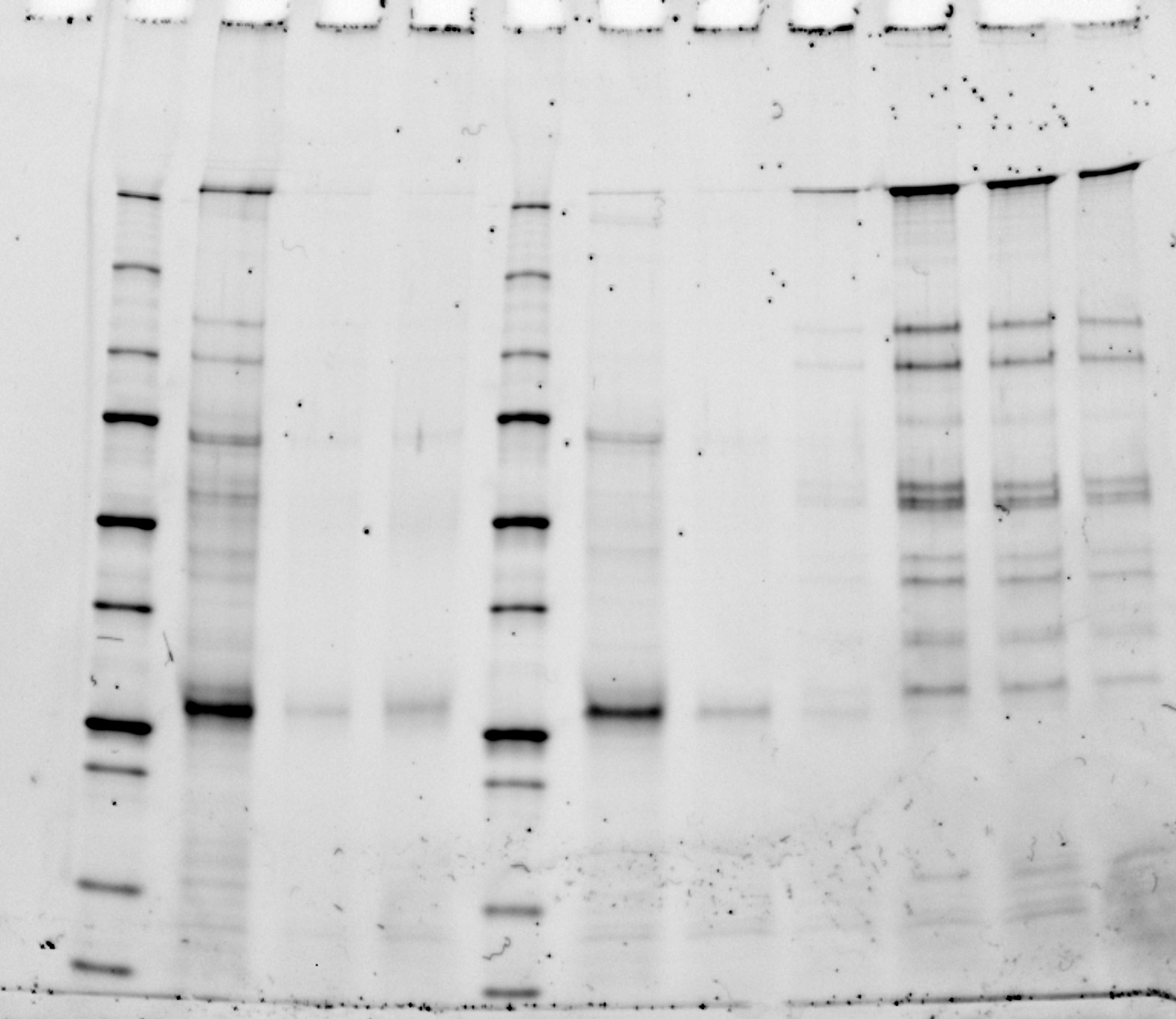

Supplement: Figure 1—figure supplement 1—source data 1. — For panel A. [file elife-81400-fig1-figsupp1-data1.zip › Figure 1-figure supplement 1-source data 1/2017_11_03 NuA4 post_IgG_TEV_CBP [Flamingo]_withoutLabels.tif]

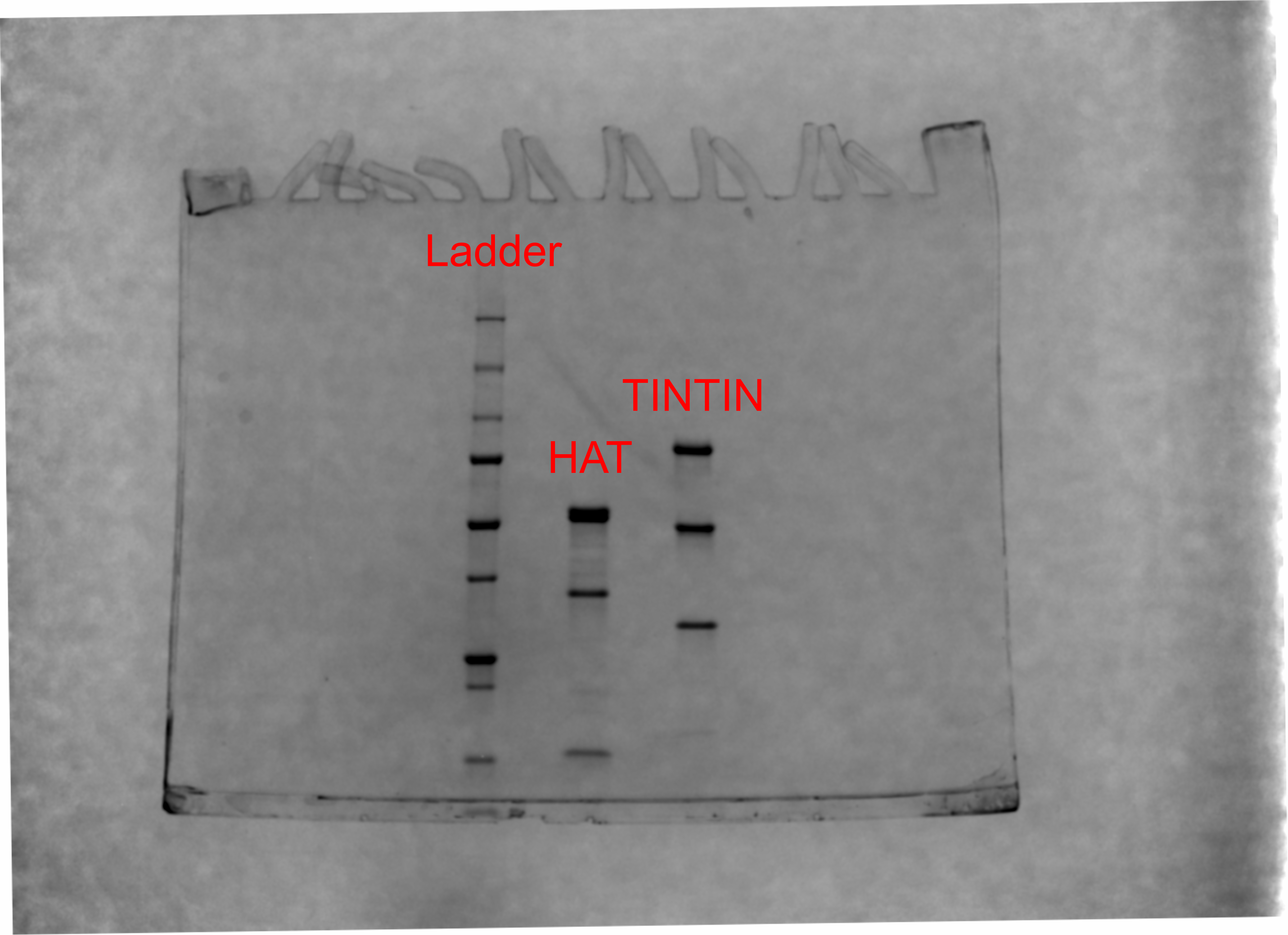

Supplement: Figure 3—source data 1. — For panel C. [file elife-81400-fig3-data1.zip › Figure 3-source data 1/2020-08-18 nuA4 HAT TINTIN Pretty Gel.tif]

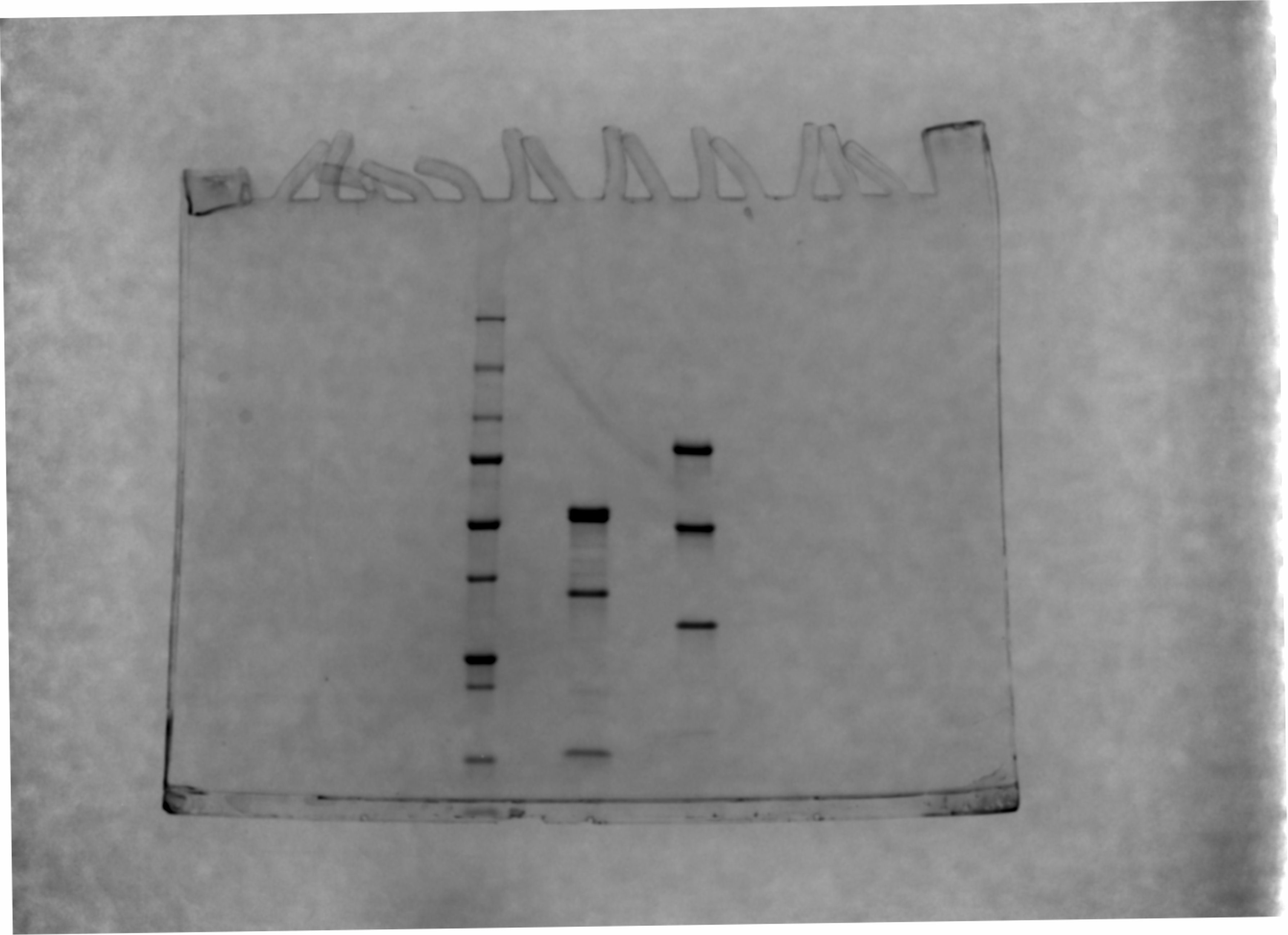

Supplement: Figure 3—source data 1. — For panel C. [file elife-81400-fig3-data1.zip › Figure 3-source data 1/2020-08-18 nuA4 HAT TINTIN Pretty Gel_withoutLabels.tif]
